# Factors associated with job satisfaction among Chinese community health workers: a cross-sectional study

**DOI:** 10.1186/1471-2458-11-884

**Published:** 2011-11-24

**Authors:** Cuixia Ge, Jialiang Fu, Ying Chang, Lie Wang

**Affiliations:** 1 Department of Social Medicine, School of Public Health, China Medical University, Shenyang, Liaoning, People's Republic of China

## Abstract

**Background:**

With the medical reform, the function of community health centres emerged to be more important recently in China. However, the health service capabilities were tremendously different between metropolitan cities and small cities. This study aims to clarify the level of job satisfaction of Chinese community health workers between a metropolitan (Shenyang) and a small city (Benxi) in Liaoning province and explore its associated factors.

**Methods:**

A cross-sectional survey was conducted from December 2009 to February 2010. A multi-stage sample was used and a total of 2,100 Chinese community health workers from the two cities completed self-administered questionnaire pertaining to job satisfaction indicated by Minnesota Satisfaction Questionnaire (MSQ), demographic characteristic and working situations, stress and job burnout. The effective response rate was 80.7%. Hierarchical regression analysis was performed to explore the related factors. All data analyses for the two cities were performed separately.

**Results:**

The averages of overall job satisfaction score of Chinese community health workers were 67.17 in Shenyang and 69.95 in Benxi. Intrinsic job satisfaction and extrinsic job satisfaction among Chinese community health workers were significantly different between Shenyang and Benxi (*p *< 0.05). In Shenyang, hierarchical regression analysis showed that the fourth model explained 36%, 32% of the variance of intrinsic and extrinsic job satisfactions, respectively. In Benxi, the fourth model explained 48%, 52% of the variance of intrinsic and extrinsic job satisfactions, respectively. Three significant predictors of intrinsic and extrinsic job satisfactions for the two cities were the two dimensions (social support and decision latitude) of stress and cynicism of burnout.

**Conclusion:**

From this study, the job satisfaction among Chinese community health workers in the two cities enjoyed a moderate level of job satisfactions, which represented they are not fully satisfied with their jobs. Community health workers in Shenyang had lower job satisfaction as compared to those in Benxi. This study strengthened the evidence that stress and burnout were important predictors of intrinsic and extrinsic job satisfactions.

## Background

China's health care system is a three-tiered system which consists of municipal and provincial hospital, district hospitals, community health centres (CHCs) in urban areas. In this system, CHCs play an important role in providing primary care to local residents, in order to link the lower and upper levels [1]. Community health workers including physicians, nurses and others are employed by CHCs to provide primary medical services. The community health centre is a system which promotes the implementation of primary public health. From October 1949 to October 1992, China was in the era of planned economy. Under the planned economy, the physicians with a college or higher education were assigned to each big hospital. Thus, there were so few physicians with a college or higher education in the community health centres. In China, barefoot doctors were the main parts of the community health workers. Generally, barefoot doctors are farmers who graduated from secondary school and received minimal basic medical and paramedical training. In Chinese culture, this means that some physicians don't have a college education in the community health centres. However, most physicians have a college education in big hospitals. Thus, the government had many methods for developing the community health centre, such as direct investment, strategic investment, government purchasing and so on. Since 1997, the Chinese urban community health care system has been rebuilt. To date, the number of CHCs has increased rapidly in China. According to the Liaoning Health statistics yearbook, there are 796 urban CHCs with nearly 10,500 community health workers in Liaoning province alone in 2008. At the end of 2007, the local government first implemented a policy, 'unlinked lines of revenue and expenditure' [2] in funding community health services in Shenyang, Liaoning Province. "Unlinked lines of revenue and expenditure" means that the government provides the funds to run the hospital, but the level of funding is not linked to the actual expenditure, including the salaries of doctors. It has brought greater convenience to local residents and reached their target of enhancing six main objectives which are medical treatment, prevention, health care, recovery, health education and family planning [3].

Job satisfaction is the extent to which people like their jobs. As a multi-dimensional complicated construct, job satisfaction includes intrinsic and extrinsic dimensions [4]. Intrinsic job satisfaction assesses the nature of the job tasks themselves such as professional development opportunities and other similar factors, while extrinsic job satisfaction assesses aspects of the working situation that are external to the job tasks such as wages, benefits and bonuses [5]. A previous study indicated that job satisfaction was associated with various factors and should not be explained by a single factor [6]. Dissatisfied health workers may be more likely to experience medical problems themselves [7] and their health has implications for stability in the health care provider workforce [8]. Some studies suggested that the factors influencing job satisfaction could be demography characteristics and working situation [9,10]. Moreover, there is a greater relationship between job satisfaction and stress [11]. It is reported that emotional exhaustion and cynicism are negatively associated with job satisfaction as well as a positively association with personal accomplishment. Also, they found that decision latitude made an important contribution to job satisfaction [12]. It is shown that job satisfaction was negatively correlated with both emotional exhaustion and cynicism in line with a slight positive correlation with personal accomplishment [13,14]. To date, most studies have focus on the local residents' satisfaction; however, according to our knowledge, no known related study has been reported clarifying the level of job satisfaction among community health workers between in a metropolitan city and a small city. In fact, few studies have specifically evaluated job satisfaction among community health workers after the implementation of the 'unlinked lines of revenue and expenditure' policy.

Overall, we hypothesized that intrinsic and extrinsic job satisfaction was associated with various factors among Chinese community health workers, such as demographic characteristic, working situation, stress and burnout. This study aims to clarify the level of job satisfaction of Chinese community health workers between a metropolitan (Shenyang) and a small city (Benxi) in Liaoning province and explore its associated factors.

## Methods

### Sample

Liaoning Province is located in northeast China, with a total of about 10,500 community health workers in 796 urban CHCs. In this study, a multi-stage sampling method was applied. Firstly, according to different city sizes, two cities (Shenyang and Benxi) were selected from Liaoning province. Shenyang is a metropolitan city and its population is about 7.2 million; Benxi in the other hand is a small city with a population of about 3.1 million. Both cities are the economic centres in Liaoning Province. Secondly, 15 CHCs were randomly selected from each city, each with a total of 70 participants. Thirdly, a total of 2,100 participants from the 30 CHCs were randomly sampled from the two cities. Each participant was informed by a written document that participation in the study was voluntary and their privacy would be strictly protected. After providing written informed consent for the conduct of the survey, a self-administered questionnaire survey was conducted among the community health workers. Trained research assistants handed the questionnaires to the participants and later collected them after completion. Finally, 1,694 participants responded and served as subjects in this study. The response rate was 80.7%.

### Questionnaire

#### Assessment of job satisfaction

The Minnesota Satisfaction Questionnaire (MSQ) [15] is a well regarded measure of job satisfaction. The 20-item short form MSQ used a 5-point scale (from 1 = very dissatisfied to 5 = very satisfied), including two dimensions: intrinsic job satisfaction and extrinsic job satisfaction. Intrinsic job satisfaction includes 12 items that refer to activity, ability utilization, achievement, and so forth. Extrinsic job satisfaction includes 6 items that refer to supervision-human relation, company policies, compensation, and so forth. Intrinsic and extrinsic job satisfactions increase with higher score. Overall job satisfaction is a total of 20 items and can be considered as a composite of all the facets of job satisfaction. The score of overall job satisfaction ranges from 20 to 100. A score of 'either very dissatisfied or dissatisfied with their job' is 40 and below; of 'dissatisfied to moderate' is from 41 to 59; of 'moderate' is 60; of 'moderate to not fully satisfied' is from 61 to 79; of 'very satisfied or satisfied' is 80 and above [4]. In this study, the Cronbach's alphas for the total job satisfaction, intrinsic job satisfaction and extrinsic job satisfaction of the MSQ were 0.93, 0.90 and 0.83, respectively.

#### Assessment of demographic characteristics

The demographic characteristics questionnaire included age, gender, educational level, marital status, sleep disorder, exercise, negative life event and chronic disease.

'Sleep disorders' was defined as 'yes' if any disorder (e.g. insomnia, sleep apnea, restless leg syndrome, and sleepwalking) had ever been diagnosed. 'Exercise' (doing exercise more than one time every week) and 'negative life events' (e.g. death of a family member, major illness of a significant other, losing a job) were both classified into 'no' and 'yes'. 'Chronic disease' was defined as 'yes' if any common chronic disease (e.g. hypertension, cardiovascular disease, diabetes, and arthritis) had ever been diagnosed [16].

#### Assessment of working situations

The working conditions questionnaire included occupation, department, job rank, weekly working time, night shift, shift work and doctor-patient relationship. 'Community health workers-patient relationship' was determined with the question 'How often have you felt distracted by patients at work?' Responses of 'never', 'rarely' or 'sometimes' were grouped as 'general degree, and 'frequently' or 'always' were grouped as 'serious degree', in line with another study [17].

#### Assessment of stress

The Chinese version of Job Content Questionnaire (JCQ) is a reliable and valid instrument for measuring stress among Chinese health workers [18]. It was used because it included a specific factor such as social support which is not available in other stress instruments. The 22-item JCQ questionnaire used a 4-point scale, including three dimensions: decision latitude, job demands and social support. Low scores on the decision latitude or social support dimensions indicated stress, as do high scores on the job demand dimension. The reliability of the JCQ was high (Cronbach's α = 0.85). The Cronbach's alphas for the subscale "decision latitude", "job demands" and "social support" were 0.64, 0.50 and 0.88 respectively. However, it was relatively low for the job demands subscale. According to national studies[18,19], the Cronbach's alpha coefficients for job demand were 0.55 in Taiwan and 0.56 in mainland China, respectively. For international studies, the alpha for the job demands subscales ranged from 0.5 to 0.7 [20,21]. Thus, our results seemed to be acceptable.

#### Assessment of burnout

The Chinese version of Maslach Burnout Inventory-General Survey (MBI-GS) is a widely using instrument for measuring burnout [22]. The 15-item MBI-GS questionnaire used a 6-point scale, including three dimensions: emotional exhaustion, cynicism and personal accomplishment. High scores on the emotional exhaustion or cynicism dimensions indicated burnout, as do low scores on the reduced personal accomplishment dimension. In our study, the Cronbach's α of the MBI-GS was 0.84. The Cronbach's alphas for the subscale "emotional exhaustion", "cynicism" and "personal accomplishment" were 0.93, 0.88 and 0.94 respectively.

### Field work

A cross-sectional survey was conducted from December 2009 to February 2010, using the self-administered interview method. The protocol of this study was approved by the Research Ethics Committee, China Medical University. All the participants enrolled voluntarily and their privacy would be strictly protected.

### Data management and statistical analysis

All qualified questionnaires were double-entered by two independent professional data processors with Epidata 3.0. If the ratio of missing data in the questionnaire was <25%, the questionnaires were considered 'qualified'; If the ratio was >25%, the questionnaire was excluded from analysis. All data was shown as number (N), percentage (%), mean, and standard deviation (SD). Correlations among job satisfaction and stress, burnout were calculated with Pearson correlation. Hierarchical regression analysis was used to explore the associated factor with intrinsic and extrinsic job satisfaction, which was dependent on demographic characteristic, working situation, stress and burnout. For the regression: we prefer presenting from step 1 to step 4. Provide data: Adj.R^2^, R^2^-changes, F, Sig. for each step and for only the variables that are significant, provide the Beta, t, and Sig. All data analyses were performed using SPSS 13.0. A two-tailed *P*-value <0.05 was defined as significant. All data analyses for the two cities were performed separately.

## Results

### Demographic characteristics and working situations of samples

The distribution of demographic variables in the two cities could be found in Table 1. 224 (22.2%) of the participants were male and 786 (77.8%) of the participants were female in Shenyang. 108(15.8%) of the participants were male and 576(84.2%) of the participants were female in Benxi. Gender, age, educational level, sleep disorders, exercise, chronic disease, occupation, department, job rank, weekly work time, community health worker-patient relationship were significantly different in the two cities (*P *< 0.05).

**Table 1:** Demographic characteristics and working situation of the samples [*N *(%)]

	Shenyang *N *= 1010	Benxi *N *= 684	*P *value
Demographic characteristics			

Gender			

male	224(22.2)	108(15.8)	<0.05

female	786(77.8)	576(84.2)	

Age group (years)			

<30	160(15.8)	155(22.7)	<0.05

30-	294(29.1)	146(21.3)	

40-	556(55.0)	383(56.0)	

Educational level			

high school or below	270(26.7)	322(47.1)	<0.05

junior college	439(43.5)	259(37.9)	

college or above	301(29.8)	103(15.0)	

Marital status			

single/widow/divorced/separated	200(19.8)	155(22.7)	ns

married/cohabitation	810(80.2)	529(77.3)	

Sleep disorders			

no	511(50.6)	266(38.9)	<0.05

yes	499(49.4)	418(61.1)	

Exercise			

no	569(56.3)	337(49.2)	<0.05

yes	441(43.7)	347(50.8)	

Chronic disease			

no	795(78.7)	483(70.6)	<0.05

yes	215(21.3)	201(29.4)	

Life events			

no	616(61.0)	414(60.5)	ns

yes	394(39.0)	270(39.5)	

Working situation			

Occupation			

physician	572(56.6)	276(40.4)	<0.05

nurse	358(35.4)	319(46.6)	

others	80(7.9)	89(13.0)	

Department			

medicine and surgery	228(22.6)	233(34.1)	<0.05

gynaecology and paediatrics	161(15.9)	50(7.3)	

paramedical units	212(21.0)	131(19.1)	

others	409(40.5)	270(39.5)	

Job rank			

low	440(43.6)	427(62.4)	<0.05

middle	487(48.2)	215(31.5)	

high	83(8.2)	42(6.1)	

Weekly work time			

≤40 h/week	749(74.2)	358(53.2)	<0.05

>40 h/week	261(25.8)	320(46.8)	

Night shift			

no	671(66.4)	448(65.5)	ns

yes	335(33.2)	236(34.5)	

Shift work			

no	682(67.5)	457(66.8)	ns

yes	328(32.5)	227(33.2)	

Community health worker-patient relationship			

general degree	927(91.8)	652(95.3)	<0.05

serious degree	83(8.2)	32(4.7)	

ns not significant

### The level of job satisfaction, stress and burnout

The mean value of the overall job satisfaction was closer to moderated than satisfied among the community health workers in Shenyang (67.17 ± 10.16) and in Benxi (69.95 ± 10.88). All aspects of the job satisfaction were significantly lower in Shenyang in comparison to Benxi (*P *< 0.05). The two dimensions (decision latitude and social support) of stress were lower in Shenyang than those in Benxi (*P *< 0.05). (Table 2).

**Table 2:** The level of job satisfaction, stress and burnout [Mean (SD)]

	Shenyang *N *= 1010	Benxi *N *= 684	*P *value
Minnesota Satisfaction Questionnaire (MSQ)			

Overall job satisfaction	67.17 ± 10.16	69.95 ± 10.88	<0.05

Intrinsic job satisfaction	40.57 ± 6.00	42.37 ± 6.43	<0.05

Extrinsic job satisfaction	19.58 ± 3.66	20.06 ± 4.02	<0.05

Job Content Questionnaire (JCQ)			

Decision Latitude	61.66 ± 8.15	62.97 ± 8.16	<0.05

Job Demands	31.70 ± 3.54	31.54 ± 3.51	ns

Social Support	24.22 ± 2.76	24.82 ± 3.06	<0.05

Maslach Burnout Inventory General Survey (MBI-GS)			

Emotional exhaustion	7.20 ± 5.49	6.88 ± 5.81	ns

Cynicism	3.55 ± 4.28	3.28 ± 4.37	ns

Personal accomplishment	24.41 ± 10.78	25.42 ± 10.50	ns

ns not significant

### Correlations among job satisfaction, stress and burnout

The correlations range from −0.014 (personal accomplishment with intrinsic job satisfaction in Benxi) to 0.483 (decision latitude with intrinsic job satisfaction in Benxi). The three subscales (decision latitude, job demands and social support) of the stress and the three subscales (emotional exhaustion, cynicism and personal accomplishment) of the burnout were correlated significantly with intrinsic and extrinsic job satisfaction in the total population, but the correlations between personal accomplishment and extrinsic job satisfaction were not significant. The three subscales of the stress and the three subscales of the burnout were related significantly to intrinsic and extrinsic job satisfactions in Shenyang. The three subscales of the stress were correlated significantly with intrinsic and extrinsic job satisfaction, but the correlations between job demands and intrinsic job satisfaction were not significant. The two subscales (emotional exhaustion and cynicism) of the burnout were related significantly to intrinsic and extrinsic job satisfaction. However, personal accomplishment was not related significantly to intrinsic and extrinsic job satisfaction (Table 3).

**Table 3:** Correlation analysis for stress, burnout and satisfaction

	Total		Shenyang		Benxi	
	**Intrinsic**	**Extrinsic**	**Intrinsic**	**Extrinsic**	**Intrinsic**	**Extrinsic**

JCQ						

Decision Latitude	0.450**	0.366**	0.419**	0.341**	0.483**	0.396**

Job Demands	−0.119**	−0.128**	−0.144**	−0.132**	−0.076	−0.121**

Social Support	0.418**	0.439**	0.402**	0.439**	0.417**	0.429**

MBI-GS						

Emotional Exhaustion	−0.301**	−0.278**	−0.301**	−0.264**	−0.293**	−0.296**

Cynicism	−0.358**	−0.314**	−0.384**	−0.336**	−0.316**	−0.281**

Personal accomplishment	0.086**	0.037	0.140**	0.099**	−0.014	−0.067

**P < 0.01, *P < 0.05

### Factors associated with job satisfaction

#### Factors with intrinsic and extrinsic job satisfaction in the total population

The main factors associated with intrinsic job satisfaction in the total population are shown in Table 4. After four steps, the demographic factors, the working situation, the stress and burnout explained 38% of the intrinsic job satisfaction. All independent variables that accounted for 35% of the extrinsic job satisfaction could be found in Table 5.

**Table 4:** The hierarchical regression analysis for intrinsic job satisfaction in the total population

Variables	Adj.R^2^	R^2 ^-changes	F	**Sig**.	Beta	t	**Sig**.
Step 1	0.03	0.03	5.85	<0.001			

Demographic factors							

Sleep disorders					−0.12	−4.09	<0.001

Exercise					0.10	3.37	0.001

Negative life events					−0.07	−2.43	0.015

Step 2	0.06	0.03	6.26	<0.001			

Demographic factors							

Sleep disorders					−0.08	−2.69	0.007

Exercise					0.10	3.67	<0.001

Working situation							

Community health worker-patient relationship					−0.16	−5.68	<0.001

Step 3	0.31	0.25	32.58	<0.001			

Demographic factors							

Exercise					0.05	2.12	0.034

Working situation							

Weekly work time					−0.05	2.15	0.032

Community health worker-patient relationship					−0.11	−4.33	<0.001

JCQ							

Decision Latitude					0.30	11.12	<0.001

Job Demands					−0.07	−2.82	0.005

Social Support					0.30	11.44	<0.001

Step 4	0.38	0.07	38.26	<0.001			

JCQ							

Decision Latitude					0.28	10.84	<0.001

Job Demands					−0.07	−2.77	0.006

Social Support					0.27	10.89	<0.001

MBI-GS							

Emotional Exhaustion					−0.07	−2.47	0.014

Cynicism					−0.24	−8.25	<0.001

**Table 5:** The hierarchical regression analysis for extrinsic job satisfaction in the total population

Variables	Adj.R^2^	R^2^-changes	F	**Sig**.	Beta	t	**Sig**.
Step 1	0.04	0.04	6.79	<0.001			

Demographic factors							

Sleep disorders					−0.12	−4.17	<0.001

Negative life events					−0.12	−4.10	<0.001

Step 2	0.06	0.02	6.19	<0.001			

Demographic factors							

Sleep disorders					−0.09	−3.02	0.003

Negative life events					−0.10	−3.47	0.001

Working situation							

Community health worker-patient relationship					−0.15	−5.05	<0.001

Step 3	0.29	0.23	30.43	<0.001			

Demographic factors							

Sleep disorders					−0.05	−1.99	0.046

Negative life events					−0.09	−3.73	<0.001

Working situation							

Community health worker-patient relationship					−0.10	−3.98	<0.001

JCQ							

Decision Latitude					0.20	7.22	<0.001

Job Demands					−0.08	−3.13	0.002

Social Support					0.37	13.98	<0.001

Step 4	0.35	0.06	34.14	<0.001			

Demographic factors							

Negative life events					−0.08	−3.35	0.001

JCQ							

Decision Latitude					0.18	6.73	<0.001

Job Demands					−0.07	−2.79	0.005

Social Support					0.35	13.65	<0.001

MBI-GS							

Emotional Exhaustion					−0.09	−2.87	0.004

Cynicism					−0.21	−7.12	<0.001

#### Factors with intrinsic and extrinsic job satisfaction in Shenyang

The findings of the hierarchical regression analysis for the intrinsic job satisfaction in Shenyang were shown in Table 6. In step 1, the demographic factors accounted for 3% of the variance. In step 2, the working situation explained an additional 3% of the variance. In step 3, stress made up an additional 22% of the variance. In the final step, the burnout accounted for an additional 8% of the intrinsic job satisfaction. The department was positively associated with the intrinsic job satisfaction in Shenyang (β = 0.09, *p *< 0.05); However, it was not associated with the extrinsic job satisfaction. All aspects of the stress were significantly associated with the intrinsic job satisfaction. As for the burnout, cynicism showed a negative beta weight (β =−0.27, *p *< 0.05), while, personal accomplishment represented a positive beta weight (β = 0.05, *p *< 0.05).

**Table 6:** The hierarchical regression analysis for intrinsic job satisfaction in Shenyang

Variables	Adj.R^2^	R^2 ^-changes	F	**Sig**.	Beta	t	**Sig**.
Step 1	0.03	0.03	4.84	<0.001			

Demographic factors							

Age group (years)					0.08	2.07	0.039

Sleep disorders					−0.11	−3.43	0.001

Exercise					0.10	3.01	0.003

Negative life events					−0.07	−2.11	0.035

Step 2	0.06	0.03	5.16	<0.001			

Demographic factors							

Educational level					0.08	2.30	0.022

Sleep disorders					−0.07	−2.24	0.025

Exercise					0.10	3.23	0.001

Working situation							

Weekly work time					−0.08	−2.40	0.017

Community health worker-patient relationship					−0.14	−4.21	<0.001

Step 3	0.28	0.22	22.66	<0.001			

Demographic factors							

Educational level					0.07	2.13	0.033

Working situation							

Department					0.08	2.84	0.005

Weekly work time					−0.07	−2.35	0.019

Community health worker-patient relationship					−0.09	−3.28	0.001

JCQ							

Decision Latitude					0.27	8.79	<0.001

Job Demands					−0.08	−2.82	0.005

Social Support					0.30	10.16	<0.001

Step 4	0.36	0.08	28.08	<0.001			

Working situation							

Department					0.09	3.41	0.001

JCQ							

Decision Latitude					0.24	8.21	<0.001

Job Demands					−0.09	−3.26	0.001

Social Support					0.28	9.91	<0.001

MBI-GS							

Cynicism					−0.27	−8.07	<0.001

Personal accomplishment					0.05	1.99	0.047

The results of the factors associated with the extrinsic job satisfaction could be found in Table 7. In the final step, negative life events, weekly work time, job demand and cynicism were significantly negatively associated with the extrinsic job satisfaction. In contrast, two subscales (decision latitude and social support) were positively related with the extrinsic job satisfaction in Shenyang.

**Table 7:** The hierarchical regression analysis for extrinsic job satisfaction in Shenyang

Variables	Adj.R^2^	R^2^-changes	F	**Sig**.	Beta	t	**Sig**.
Step 1	0.03	0.03	4.29	<0.001			

Demographic factors							

Sleep disorders					−0.09	−2.86	0.004

Negative life events					−0.13	−4.06	<0.001

Step 2	0.05	0.02	4.63	<0.001			

Demographic factors							

Negative life events					−0.11	−3.52	<0.001

Working situation							

Weekly work time					−0.11	−3.20	0.001

Community health worker-patient relationship					−0.13	−3.87	<0.001

Step 3	0.27	0.22	21.37	<0.001			

Demographic factors							

Negative life events					−0.11	−3.72	<0.001

Working situation							

Weekly work time					−0.10	−3.39	0.001

Community health worker-patient relationship					−0.09	−3.16	0.002

JCQ							

Decision Latitude					0.18	5.93	<0.001

Job Demands					−0.08	−2.67	0.008

Social Support					0.36	12.25	<0.001

Step 4	0.32	0.05	23.47	<0.001			

Demographic factors							

Negative life events					−0.10	−3.55	<0.001

Working situation							

Weekly work time					−0.09	−3.00	0.003

JCQ							

Decision Latitude					0.16	2.28	<0.001

Job Demands					−0.08	2.90	0.004

Social Support					0.35	12.07	<0.001

MBI-GS							

Cynicism					−0.23	−6.45	<0.001

#### Factors with intrinsic and extrinsic job satisfaction in Benxi

The main factors related with the intrinsic job satisfaction in Benxi were showed in Table 8. In the final step, all variables accounted for 48% of the variance. The education level and cynicism were negatively associated with the intrinsic job satisfaction and the beta weights (β) were −0.13 and −0.20, respectively. On the contrary, two subscales (decision latitude and social support) were positively related with the intrinsic job satisfaction and the beta weights (β) were 0.36 and 0.24, respectively.

**Table 8:** The hierarchical regression analysis for intrinsic job satisfaction in Benxi

Variables	Adj.R^2^	R^2^-changes	F	**Sig**.	Beta	t	**Sig**.
Step 1	0.09	0.09	4.42	<0.001			

Demographic factors							

Educational level					−0.19	−3.08	0.002

Sleep disorders					−0.17	−2.71	0.007

Chronic disease					−0.14	−2.35	0.019

Step 2	0.14	0.05	3.93	<0.001			

Demographic factors							

Educational level					−0.19	−2.92	0.004

Sleep disorders					−0.16	−2.63	0.009

Working situation							

Community health worker-patient relationship					−0.21	−3.41	0.001

Step 3	0.43	0.29	12.21	<0.001			

Demographic factors							

Educational level					−0.15	−2.88	0.004

Working situation							

Community health worker-patient relationship					−0.13	−2.49	0.013

JCQ							

Decision Latitude					0.35	5.76	<0.001

Social Support					0.29	5.04	<0.001

Step 4	0.48	0.05	12.54	<0.001			

Demographic factors							

Educational level					−0.13	−2.56	0.011

JCQ							

Decision Latitude					0.36	6.20	<0.001

Social Support					0.24	4.30	<0.001

MBI-GS							

Cynicism					−0.20	−3.23	0.001

As for the extrinsic job satisfaction, the main factors could be found in Table 9. In the final step, all variables accounted for 52% of the variance. The main factor associated with extrinsic job satisfaction was social support (β = 0.32, *p *< 0.05).

**Table 9:** The hierarchical regression analysis for extrinsic job satisfaction in Benxi

Variables	Adj.R^2^	R^2 ^-changes	F	**Sig**.	Beta	t	**Sig**.
Step 1	0.09	0.09	4.43	<0.001			

Demographic factors							

Educational level					−0.19	−3.04	0.003

Sleep disorders					−0.22	−3.55	<0.001

Step 2	0.17	0.08	4.66	<0.001			

Demographic factors							

Educational level					−0.14	−2.23	0.026

Sleep disorders					−0.20	−3.34	0.001

Working situation							

Department					−0.20	−3.32	0.001

Weekly work time					0.13	2.08	0.039

Night shift					−0.22	−2.77	0.006

Community health worker-patient relationship					−0.20	−3.22	0.001

Step 3	0.44	0.27	12.60	<0.001			

Demographic factors							

Educational level					−0.11	−1.98	0.048

Sleep disorders					−0.13	−2.53	0.012

Working situation							

Occupation					0.13	2.48	0.014

Department					−0.17	−3.44	0.001

Weekly work time					0.13	2.56	0.011

Night shift					−0.18	−2.59	0.010

Community health worker-patient relationship					−0.13	2.56	0.011

JCQ							

Decision Latitude					0.21	3.52	0.001

Social Support					0.37	6.63	<0.001

Step 4	0.52	0.08	14.76	<0.001			

Working situation							

Occupation					0.12	2.44	0.02

Department					−0.17	−3.64	<0.001

Weekly work time					0.14	2.87	0.005

Night shift					−0.13	−2.14	0.033

JCQ							

Decision Latitude					0.23	4.18	<0.001

Social Support					0.32	5.97	<0.001

MBI-GS							

Emotional Exhaustion					−0.18	−2.85	0.005

Cynicism					−0.18	−3.05	0.003

## Discussion

### Job satisfaction

In this study, the results indicated that Chinese community health workers in the two cities enjoyed a moderate level of job satisfaction, which is in line with recent studies [23,24]. Nine percent were either very dissatisfied or dissatisfied with their jobs; 16.9% were dissatisfied to moderate; 8.2% were moderate; 59.1% were moderate to not fully satisfied, while 14.9% were very satisfied or satisfied with their jobs.

This showed that they are not fully satisfied with their job. Another finding is that the community health workers in Shenyang had lower job satisfaction as compared to Benxi. It was possible that the community health workers undergo more stress, burnout and competition in the big city (Shenyang) than those in the small city (Benxi). The special policy "unlinked lines of revenue and expenditure" which is a widely used model of payment in order to reduce health expenditures was first implemented by the Shenyang local government at the end of 2007. Community residents become the biggest beneficiaries, while community health workers' personal income and the efficiency of health care service have been weakened. It also increased the stress and competition with the health care work force. Specially, the amount of outpatient cases in the CHCs in Shenyang increased rapidly, leading to long-term overload and burnout among the community health workers. All of the above might subsequently induce lower satisfaction among the community health workers. Thus, a social support system for community health workers might be given first priority by the Shenyang local government.

### Factor with intrinsic and extrinsic job satisfaction in two cities

#### Demography characteristics and job satisfaction

There seems not to be a clear trend between educational level and job satisfaction. Some researches considered that the higher educational level was linked with higher level of job satisfaction [25,26]. On the contrary, other studies showed that job satisfaction are lower among better educated population [27]. In this study, we found that educational level was a negative predictor of the intrinsic job satisfaction in Benxi. The Benxi local government gives more promotion and provides continuing education chance for lower educated community health workers, thus they may have higher intrinsic job satisfaction, such as ability utilization and achievement. It is noted that the Benxi local government should focus on the better educated community health workers, provide them more opportunities to do something that makes use of their abilities, improve their feeling of accomplishment from the job and so forth. Negative life events, such as death of a family member, major illness of a significant other, losing a job played an important role in triggering low extrinsic job satisfaction from our study.

#### Working situations and job satisfaction

Working characteristics might be appropriate for community health workers' needs and expectations. It consisted of factors such as weekly working time and night shift, which affected workers' productivity. Community health workers with low job satisfaction have been linked to heavy workload, mostly due to lacking enough resting time and long working hours [28,29]. Thus, more attention should be paid to these factors and modifications made to the workload in order to increase the workers' intrinsic and extrinsic job satisfaction. Night shift is the hardest because of the sleeping rhythm and because of having to work alone during the shift. In this study, we found that night shift is a negative predictor of extrinsic job satisfaction in Benxi, in line with other study [30]. In Benxi, the extra reward will be paid from the CHCs if one does additional work, e.g. having the night shift, working more than 40 hours a week, as shown in this analysis, have increased the community health worker's extrinsic job satisfaction.

#### Stress and job satisfaction

Stress in our study was the highest rated aspects contributing to the community health workers' job satisfaction. Previous studies indicated that good relations with co-workers and having the support of superiors mostly created a feeling of satisfaction, social support was the most consistent and potent predictors of job satisfaction [24,31], similar to the finding of our study. This is reflected in this study that published social networks serves as a resource that increased intrinsic and extrinsic job satisfaction. According to Nguyen, Taylor and Bradley [32], decision latitude has a significant impact on job satisfaction. The relationship is that more autonomy is associated with greater job satisfaction. The main trend observed in this study is that as decision latitude increases, community health workers report a high degree of job satisfaction, the same as other study [33]. Conversely, high job demand as described by community health workers may also be a contributory factor for lower intrinsic job satisfaction (i.e. the feeling of accomplishment from the job) and extrinsic job satisfaction (i.e. pay and the amount of work) [34]. Thus, more attention should be paid to community health workers' stress in order to increase their job satisfaction, such as fostering a good relationship between superior and subordinate, giving more chance and freedom to do the job and so forth.

#### Burnout and job satisfaction

The results of correlation analysis showed that negative correlation of job satisfaction with emotional exhaustion and cynicism, and the positive correlation with reduced personal accomplishment, which are consistent with many other studies [35,36]. The consistency indicated that our findings have international value. In a previous study, it is not clear whether job dissatisfaction caused burnout or whether burnout causes job dissatisfaction [37]. Some researchers suggested that a protective factor of burnout may be job satisfaction [12,36]. However, the hierarchical regression results of our study showed that three dimensions of burnout (i.e. emotional exhaustion, cynicism and personal accomplishment) [38,39] were associated with job satisfaction in Shenyang; two dimensions of burnout (i.e. emotional exhaustion and cynicism) were associated with job satisfaction in Benxi; cynicism was associated with both dimensions of job satisfaction (i.e. intrinsic and extrinsic job satisfaction) in the two cities. It indicated that burnout is a predictor of job satisfaction. According to Chinese Confucianism, subordinate may indirectly express their satisfaction/dissatisfaction because subordinate must obey superior. Thus, local government should focus on relieving the community health workers' burnout; Specially, Shenyang government should pay attention to give more chance to personal development and hence the personal accomplishment in order to increase the intrinsic job satisfaction, such as the chance to be 'somebody' in the community, doing things in line with one's conscience, the opportunity to tell others what to do, ability utilization and creativity. This would likely improve work efficiency and increase job satisfaction.

## Limitations

In this study, there were three limitations. First, the cross-section design with job satisfaction. It was difficult to establish causal conclusion and the longitudinal survey might be carried out to confirm the causal conclusion in our future study. Second, the measurements were performed by self-administrated method. Then, the common method bias and self-administrated bias might have an impact on the results. Finally, it is possible that the community health workers were more interested in examining their job satisfaction in big city than those in small city. Therefore, it might be due to the differences in the response rate in the two cities.

## Conclusion

From this study, the job satisfaction among Chinese community health workers within the two cities varies from moderately satisfied to not fully satisfied. Community health workers had lower job satisfaction in Shenyang in comparison to Benxi. This study strengthened the evidence that stress and burnout were negatively associated with higher job satisfaction.

## Competing interests

The authors declare that they have no competing interests.

## Authors' contributions

CXG assisted with the survey, completed the statistical analyses and drafted the manuscript. JLF assisted with the study, and XSY assisted with the survey and data analyses. YC participated in the design of the study. LW conceived of the study, and participated in its design and coordination and helped to draft the manuscript. All authors read and approved the final manuscript.

## Pre-publication history

The pre-publication history for this paper can be accessed here:

http://www.biomedcentral.com/1471-2458/11/884/prepub

## References

[B1] Meng QY Yuan J Jing LM Zhang JH Mobility of primary health care workers in China Hum Resour Health 2009 7 24 10.1186/1478-4491-7-24 19292911 PMC2661043

[B2] Bao Y Investigating two lines management mechanism of receipts and expenditures and perfecting the model of community health service with Public-spirited and Fair in Chinese [in Chinese] Appl J Gen Pract 2007 5 95 96

[B3] Li Y Cao J Lin H Li D Wang Y He J Community health needs assessment with precede-proceed model: a mixed methods study BMC Health Serv Res 2009 9 181 10.1186/1472-6963-9-181 19814832 PMC2770049

[B4] Sharp TP Job satisfaction among psychiatric registered nurses in New England J Psychiatr Ment Health Nurs 2008 15 374 378 10.1111/j.1365-2850.2007.01239.x 18454822

[B5] Hirschfeld RR Validity studies. Does revising the intrinsic and extrinsic subscales of the Minnesota Satisfaction Questionnaire Short Form make a difference? Educ Psychological Meas 2000 6 255 270

[B6] Blegen MA Nurses' job satisfaction: a meta-analysis of related variables Nurs Res 1993 42 36 41 8424066

[B7] Sundquist J Johannsson SE High demand, low control, and impaired general health: working conditions in a sample of Swedish general practitioners Scand J Public Health 2000 28 123 131 10954139 10.1177/140349480002800208

[B8] Fahrenkopf AM Sectish TC Barger LK Sharek PJ Lewin D Chiang VW Edwards S Wiedermann BL Landrigan CP Rates of medication errors among depressed and burnt out residents: prospective cohort study BMJ 2008 336 488 491 10.1136/bmj.39469.763218.BE 18258931 PMC2258399

[B9] Okerlund VW Jackson PB Parsons RJ Factors affecting recruitment of physical therapy personnel in Utah Phys Ther 1994 74 177 184 8290622 10.1093/ptj/74.2.177

[B10] Freeborn DK Satisfaction, commitment, and psychological well-being among HMO physicians West J Med 2001 174 13 18 10.1136/ewjm.174.1.13 11154654 PMC1071220

[B11] Kaarna M Põlluste K Lepnurm R Thetloff M The progress of reforms: job satisfaction in a typical hospital in Estonia Int J Qual Health Care 2004 16 253 61 10.1093/intqhc/mzh042 15150157

[B12] Ramirez AJ Graham J Richards MA Cull A Gregory WM Mental health of hospital consultants: the effects of stress and satisfaction at work Lancet 1996 347 724 728 10.1016/S0140-6736(96)90077-X 8602002

[B13] Brookings JB Bolton B Brown CE McEvoy A Self-reported job burnout among female human service professionals J Occup Behav 1985 6 143 150 10.1002/job.4030060205

[B14] Maslach C Jackson S Maslach Burnout Inventory Manual 1986 Palo Alto: CA: Consulting Psychologists Press

[B15] Weiss DJ Dawis RV England GW Lofquist LH Minnesota studies in vocational rehabilitation Man Minnesota Satisf Quest 1967 Industrial Relations Center, Minneapolis University of Minnesota

[B16] Sun W Watanabe M Tanimoto Y Shibutani T Kono R Saito M Usuda K Kono K Factors associated with good self-rated health of non-disabled elderly living alone in Japan: a cross-sectional study BMC Public Health 2007 7 297 10.1186/1471-2458-7-297 17949511 PMC2186322

[B17] Shen HC Cheng YW Tsai PJ Lee SHS Guo YL Occupational stress in nurses in psychiatric institutions in Taiwan J Occup Health 2005 47 218 225 10.1539/joh.47.218 15953843

[B18] Li J Yang W Liu P Xu Z Cho SI Psychometric evaluation of the Chinese (mainland) version of Job Content Questionnaire: a study in university hospitals Ind Health 2004 42 260 267 10.2486/indhealth.42.260 15128178

[B19] Cheng Y Luh WM Guo YL Reliability and validity of the Chinese version of the Job Content Questionnaire in Taiwanese workers Int J Behav Med 2003 10 15 30 10.1207/S15327558IJBM1001_02 12581945

[B20] Landsbergis P Kawakami N Brisson C JCQ scale validity: summary of results for decision latitude, psychological demands, and social support from the JCQ Workshop The 3rd International Conference on Work Environment and Cardiovascular Diseases 2002 Duesseldorf, Germany

[B21] Kawakami N Houtman I Tsustumi A Borg V Kiristensen T Assessment of psychological job demands: review and future directions Job Content Questionnaire (JCQ) workshop 2003 Toronto, Canada

[B22] Li CP S K The influence of distributive justice and procedural justice on job burnout [in Chinese] Acta Psychologica Sinica 2003 35 677 684

[B23] Kebriaei A Moteghedi MS Job satisfaction among community health workers in Zahedan District, Islamic Republic of Iran East Mediterr Health J 2009 S,15 1156 1163 20214129

[B24] Ofili AN Asuzu MC Isah EC Ogbeide O Job satisfaction and psychological health of doctors at the University of Benin Teaching Hospital Occup Med (Lond) 2004 54 400 403 10.1093/occmed/kqh081 15347778

[B25] Rambur B McIntosh B Palumbo MV Reinier K Education as a determinant of career retention and job satisfaction among registered nurses J Nurs Scholarsh 2005 37 185 192 10.1111/j.1547-5069.2005.00031.x 15960064

[B26] Ingersoll GL Olsan T Drew-Cates J DeVinney BC Davies J Nurses' job satisfaction, organizational commitment, and career intent J Nurs Adm 2002 32 250 263 10.1097/00005110-200205000-00005 12021566

[B27] Robinson S Murrells T Clinton M Highly qualified and highly ambitious: implications for workforce retention of realising the career expectations of graduate nurses in England Human Resour Manage J 2006 16 287 312 10.1111/j.1748-8583.2006.00015.x

[B28] Kebriaei A Moteghedi MS Job satisfaction among community health workers in Zahedan District, Islamic Republic of Iran East Mediterr Health J 2009 15 1156 1163 20214129

[B29] Rambur B Palumbo MV McIntosh B Mongeon J A statewide analysis of RNs' intention to leave their position Nurs Outlook 2003 51 182 188 10.1016/S0029-6554(03)00115-5 12949479

[B30] Norbeck JS Perceived job stress, job satisfaction, and psychological symptoms in critical care nursing Res Nurs Health 1985 8 253 259 10.1002/nur.4770080307 3852360

[B31] Baruch-Feldman C Brondolo E Ben-Dayan D Schwartz J Sources of social support and burnout, job satisfaction, and productivity J Occup Health Psychol 2002 7 84 93 11827236 10.1037//1076-8998.7.1.84

[B32] Schmidt SH Meijman TF Scholten A van Oel CJ Oort-Marburger D Factors contributing to job satisfaction following rehabilitation for musculoskeletal impairments J occup Rehabil 1993 3 213 222 10.1007/BF01097431 24243436

[B33] Kluger MT Townend K Laidlaw T Job satisfaction, stress and burnout in Australian specialist anaesthetists Anaesth 2003 58 339 345 10.1046/j.1365-2044.2003.03085.x 12648115

[B34] Bought P Pears J Evaluating the influence of the type of social support on job satisfaction and work related psychological well-being Int J Organ Behav 2004 8 472 485

[B35] Piko BF Burnout, role conflict, job satisfaction and psychosocial health among Hungarian health care staff: a questionnaire survey Int J Nurs Stud 2006 43 311 318 10.1016/j.ijnurstu.2005.05.003 15964005

[B36] Visser MR Smets EM Oort FJ De Haes HC Stress, satisfaction and burnout among Dutch medical specialists CMAJ 2003 168 271 275 12566331 PMC140468

[B37] Zedeck S Maslach C Mosier K Skitka L Affective response to work and quality of family life: Employee and spouse perspectives J Soc Behav Pers 1988 3 135 157

[B38] Maslach C Schaufeli WB Leiter MP Job burnout Annu Rev Psychol 2001 52 397 422 10.1146/annurev.psych.52.1.397 11148311

[B39] Grossi EL Berg BL Stress and job dissatisfaction among correctional officers: An unexpected finding Int J Offender Ther Comp Criminol 1991 35 73 81 10.1177/0306624X9103500107

